# Person centred approaches to learning hold a potential for a mature depth of understanding and engagement as opposed to the traditional 'transmission of knowledge' approach to learning

**DOI:** 10.1192/bjo.2021.680

**Published:** 2021-06-18

**Authors:** Catherine Hayes, Adrian Heald

**Affiliations:** 1 University of Nottingham; 2 University of Manchester, Salford Royal Hospital

## Abstract

**Aims:**

Do students experience a person-centred experiential (PCE) approach to learning in a university context differently to transmitted knowledge learning from prior education, and if so, how?

**Background:**

The person-centred approach, as defined and developed by Carl Ransom Rogers, remains on the margins of practice in the UK. The approach sustains a non-medical stance. All of the Improving Access to Psychological Therapies Person Centred Experiential Counselling (APT PCEC) workforce require a qualification in person-centred experiential counselling. In order to attune to Roger's hypothesis regarding the conditions required in order to facilitate psychological growth, person-centred learning is a principle stance.

Researching experiences of PCE learning through anonymous feedback from students attending different levels of training (BA, MA and post qualification PCE-Counselling for Depress (CfD) License) is an initial test of the hypothesis .

Counselling education in the UK is increasingly highly standardised and driven by competency frameworks. This work begins to uncover person-centred students’ evaluation of undertaking person-centred qualifications. Modules and continuing professional practice were constructed to facilitate a person-centred learning environment wherein the curriculum was designed by students or the experiential aspect of the learning drove the agenda

**Method:**

The sample was made up of (N = 30) students. 8 students were studying for a Master's degree in person-centred experiential psychotherapy, 10 students were studying a BA in humanistic psychotherapy, 12 students were attending a mandatory IAPT Continuous Professional Development (CPD) training in PCE therapy. The evaluation responses were subject to a thematic analysis.

**Result:**

The emerging themes parallel each other and indicate that degree students were very aware of the difference from their previous learning experience in education.

68% of MA Students experienced psychological maturation through the process of training.

83% of BA students became more agentic in their approach to learning.

83% IAPT therapists noticed the nurturing, compassion and humane approach to the learning, despite the mandatory nature of the offer and empowered them in regards to their non-medical stance within an NHS context.

**Conclusion:**

Our findings point to the significance and impact of person-centred learning for person-centred psychotherapists’ development during and post-qualification. Implications can be drawn in regards to engaging with person-centred learning in public sector and health contexts.

Person centred approaches to learning hold a potential for a mature depth of understanding and engagement as opposed to the traditional ‘transmission of knowledge’ approach to learning.

